# Crystal structure and Hirshfeld surface analysis of 4-(3-meth­oxy­phen­yl)-2,6-di­phenyl­pyridine

**DOI:** 10.1107/S2056989022007812

**Published:** 2022-08-23

**Authors:** Dong Cheng, Xiang-Zhen Meng, Fuyu Tian, Dong Yan, Xiaofei Wang, Xueli Qian, Junnan Wang

**Affiliations:** a Department of Chemical and Material Engineering, Chaohu College, Chaohu, People’s Republic of China; University of Kentucky, USA

**Keywords:** crystal structure, C—H⋯π inter­actions, van der Waals inter­actions, Hirshfeld surface

## Abstract

The title compound, C_24_H_19_NO, was obtained *via* the reaction of (1*E*,2*E*)-3-(3-meth­oxy­phen­yl)-1-phenyl­prop-2-en-1-one with ethyl 2-oxo­propano­ate, using NH_4_I as a catalyst. The compound crystallizes in the monoclinic space group *I*2/*a*. In the mol­ecule, the four rings are not in the same plane, the pyridine ring being inclined to the benzene rings by 17.26 (6), 56.16 (3) and 24.50 (6)°. In the crystal, mol­ecules are linked by C—H⋯π inter­actions into a three-dimensional network. To further analyse the inter­molecular inter­actions, a Hirshfeld surface analysis was performed. Hirshfeld surface analysis indicates that the most abundant contributions to the crystal packing are from H⋯H (50.4%), C⋯H/H⋯C (37.9%) and O⋯H/H⋯O (5.1%) inter­actions.

## Abstract 2

The title compound was obtained *via* the reaction of (1*E*,2*E*)-3-(3-meth­oxy­phen­yl)-1-phenyl­prop-2-en-1-one with ethyl 2-oxo­propano­ate, using NH_4_I as a catalyst. In the mol­ecule, the four rings are not in the same plane, the pyridine ring being inclined to the benzene rings by 17.26 (6), 56.16 (3) and 24.50 (6)°. In the crystal, mol­ecules are linked by C—H⋯π inter­actions into a three-dimensional network.

## 1. Chemical context

Substituted pyridines are privileged scaffolds in medicinal chemistry and are versatile building blocks for the construction of natural products (Haghighijoo *et al.*, 2020 ▸; Gujjarappa *et al.*, 2020 ▸; Nirogi *et al.*, 2015 ▸; De Rycke *et al.*, 2011 ▸; Chan *et al.*, 2010 ▸; Bora *et al.*, 2010 ▸), Accordingly, great effort has been devoted to developing efficient approaches to these scaffolds (Guin *et al.*, 2020 ▸; Wu *et al.*, 2019 ▸; Pandolfi *et al.*, 2017 ▸; Shen *et al.*, 2015 ▸). Ketoxime acetates have been demonstrated to be exceptionally advantaged and versatile building blocks for the synthesis and derivatization of nitro­gen-containing heterocycles through N—O bond cleavage (Zhang *et al.*, 2020 ▸; Mao *et al.*, 2019 ▸; Xie *et al.*, 2018 ▸). Thus far, many synthetic approaches have been developed to access nitro­gen-containing heterocycles through ketoxime acetates under metal-free conditions. For example, Duan *et al.* (2020 ▸) have successfully developed the NH_4_I-triggered formal [4 + 2] annulation of α,β-unsaturated ketoxime acetates with *N*-acetyl enamides, providing efficient access to valuable highly substituted pyridines in moderate to good yields. Gao *et al.* (2018 ▸) have developed a facile and efficient I_2_-triggered [3 + 2 + 1] annulation of aryl ketoxime acetates and 3-formyl­indoles to produce diverse 3-(4-pyrid­yl)indoles that are challenging to prepare by traditional methods. Given this background, we report herein the synthesis and crystal structure of the title compound, which was synthesized by NH_4_I-triggered annulation of α,β-unsaturated ketoxime acetates.

## 2. Structural commentary

**Figure 1:**
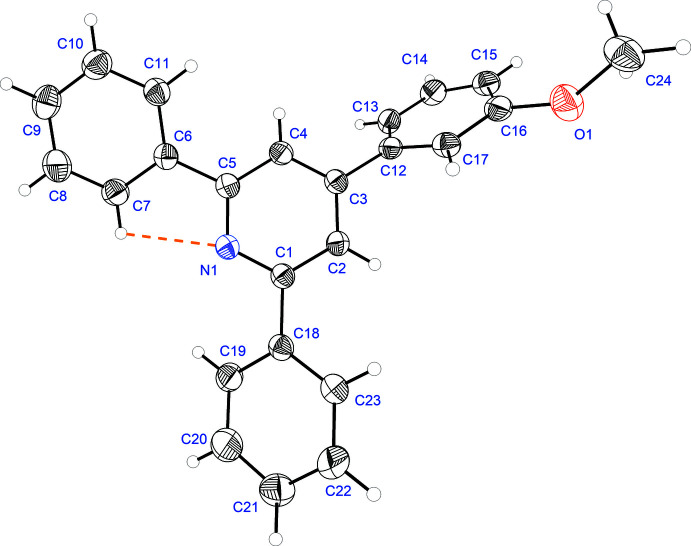
The mol­ecular structure of the title compound, with the atom labelling and displacement ellipsoids drawn at the 50% probability level. H atoms are shown as small circles of arbitrary radii.

The title compound crystallizes in the monoclinic crystal system in space group *I*2/*a*. Its mol­ecular structure is shown in Fig. 1 ▸. The meth­oxy group lies close to the mean plane of the C12–C17 phenyl ring, as indicated by the C17—C16—O1—C24 torsion angle of −170.59 (10)°, and atom C24 deviating by 0.250 (2) Å from the mean plane through the C12–C17 ring. In the mol­ecule, the four rings are not in the same plane, the pyridine ring being inclined to the C6–C11, C12–C17 and C18–C23 benzene rings by 17.26 (6), 56.16 (3) and 24.50 (6)°, respectively. There is a strong intra­molecular hydrogen bond (C7—H7⋯N1; Table 1 ▸), forming an *S*(5) ring motif. 
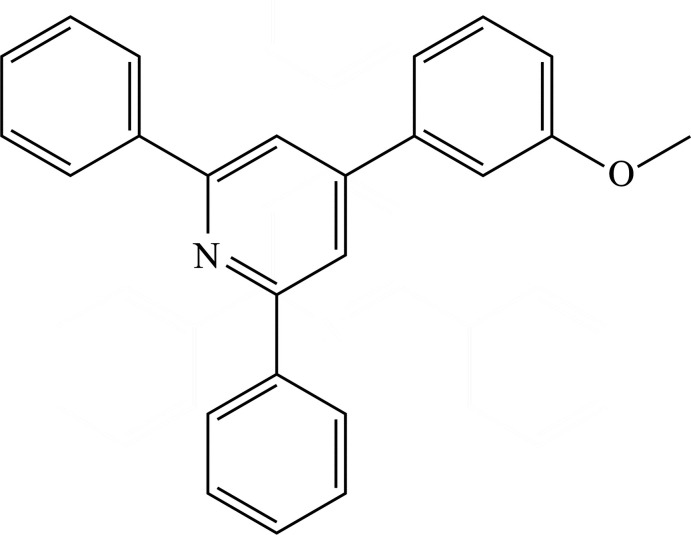


## 3. Supra­molecular features

**Figure 2:**
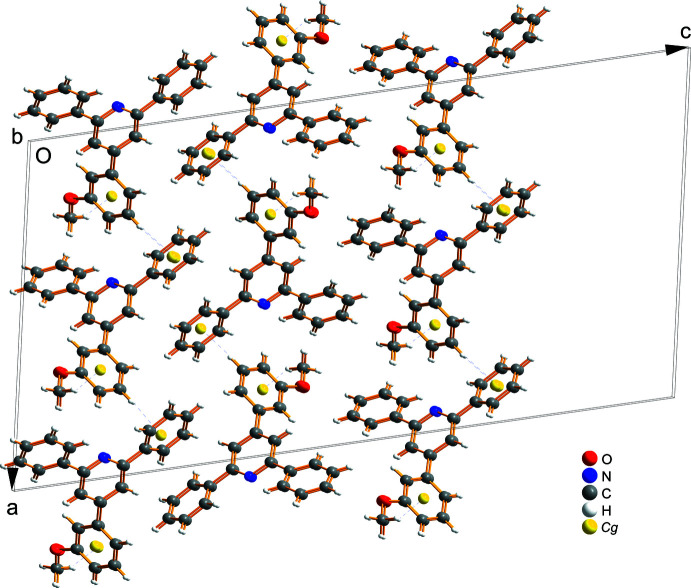
A packing diagram of the title compound. The C—H⋯π inter­actions are shown as dashed lines. Yellow spheres denoted *Cg* represent the centroids of the 3-meth­oxy­phenyl rings.

In the crystal (Fig. 2 ▸), the mol­ecules are linked by weak C—H⋯π inter­actions (C14—H14⋯*Cg*2^i^ and C24—H24⋯*Cg*3^ii^, *Cg*2 and *Cg*3 are the centroids of the C6–C11 and C12–C17 rings, respectively, symmetry codes as in Table 1 ▸). The C24—H24⋯*Cg*3 inter­actions generate stacks along the *b*-axis direction. These stacks are linked by the C14—H14⋯*Cg*2 inter­actions. The packing is strengthened by van der Waals inter­actions between parallel mol­ecular layers.

**Figure 3:**
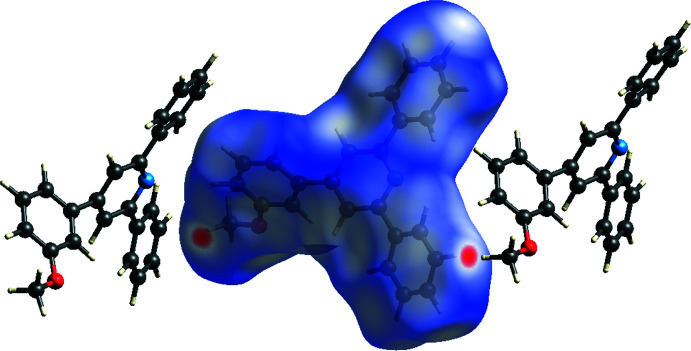
The Hirshfeld surface mapped over *d*_norm_ together with two adjacent mol­ecules.

**Figure 4:**
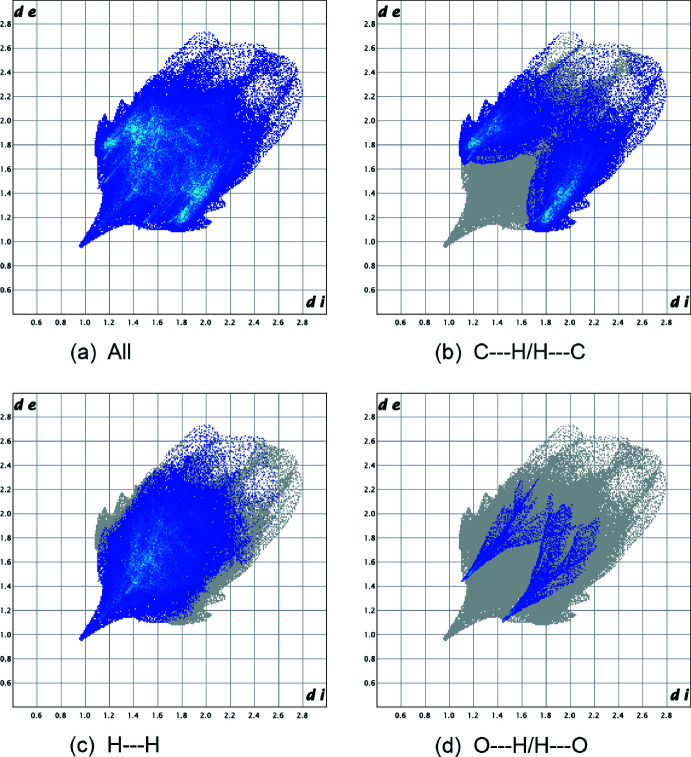
Fingerprint plots for the title mol­ecule: (*a*) all inter­molecular inter­actions, (*b*) C⋯H/H⋯C inter­actions, (*c*) H⋯H inter­actions and (*d*) O⋯H/H⋯O inter­actions.

In order to investigate the inter­molecular inter­actions in a visual manner, a Hirshfeld surface analysis was performed using *Crystal Explorer* (Spackman & Jayatilaka, 2009 ▸; Turner *et al.*, 2017 ▸). Fig. 3 ▸ shows the *d*_norm_ surface together with two adjacent mol­ecules. The bright-red spots on the Hirshfeld surface mapped over *d*_norm_ correspond to H24*B*⋯H20 (*x* − 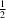 , 2 − *y*, *z*) close contacts. Fig. 4 ▸ *a* is the fingerprint plot showing all inter­molecular inter­actions while Fig. 4 ▸ *b*–*d* show these resolved into C⋯H/H⋯C (37.9%), H⋯H (50.4%) and O⋯H/H⋯O (5.1%) contributions, respectively. As a result, van der Waals inter­actions are dominant in the crystal packing.

## 4. Database survey

A search of the Cambridge Structural Database (Version 2021.1; Groom *et al.*, 2016 ▸) for the 2,4,6-tri­phenyl­pyridine moiety revealed seven structures closely related to the title compound, *viz*. 4-(4-fluoro­phen­yl)-2,6-di­phenyl­pyridine [(I) SURGER01; Zhang *et al.*, 2021 ▸], 4-[4-(azido­meth­yl)phen­yl]-2,6-di­phenyl­pyridine [(II) DOCLIT; Cheng *et al.*, 2019 ▸], 4-(4-chloro­phen­yl)-2,6-di­phenyl­pyridine [(III) GISGEV; Lv & Huang, 2008 ▸], 2,4,6-tri­phenyl­pyridine [(IV) HEVVAF, Ondráček *et al.*, 1994 ▸; HEVVAF01, Ren *et al.*, 2011 ▸; HEVVAF02, Mao *et al.*, 2017 ▸], 2-(4-methyl­phen­yl)-4,6-di­phenyl­pyridine [(V) REMHOJ; Stivanin *et al.*, 2017 ▸], 4-(4-bromo­phen­yl)-2,6-di­phenyl­pyridine [(VI) AJEZOF; Cao *et al.*, 2009 ▸], 4-(2,6-di­phenyl­pyridin-4-yl) phenol [(VII) KIDBIL; Kannan *et al.*, 2018 ▸].

As in the title compound, in (I), (II), (III), (IV) and (V), C—H⋯π (ring) inter­actions connect the mol­ecules, forming tri-periodic networks. In (VI), mol­ecules are linked by weak inter­molecular C—H⋯Br hydrogen bonds, and weak inter­molecular C—H⋯π (ring) inter­actions are also observed. In (VII), mol­ecules are linked by weak inter­molecular C—H⋯O hydrogen bonds, and there are also weak inter­molecular C—H⋯π (ring) inter­actions.

## 5. Synthesis and crystallization

(1*E*,2*E*)-3-(3-Meth­oxy­phen­yl)-1-phenyl­prop-2-en-1-one (3.0 mmol), ethyl 2-oxo­propano­ate (0.3 mmol), NH_4_I (0.22 g, 0.15 mmol) and NaHSO_3_ (0.31 g, 3.0 mmol) were loaded into a 20 mL tube under an N_2_ atmosphere. The solvent toluene (15 mL) was added into the tube by syringe. The reaction mixture was stirred at 373 K for 12 h. Upon completion of the reaction, the mixture was then allowed to cool down to room temperature and flushed through a short column of silica gel with EtOAc (15 mL). After rotary evaporation, the residue was purified by column chromatography on silica gel (petroleum ether/EtOAc) to give the product as a white solid. Part of the purified product was redissolved in petroleum ether/ethyl acetate and colourless crystals suitable for X-ray diffraction were formed after slow evaporation for several days. Spectroscopic data: ^1^H NMR (600 MHz, CDCl_3_) *δ* 8.20 (*d*, *J* = 7.8 Hz, 4H), 7.87 (*s*, 2H), 7.53–7.50 (*m*, 4H), 7.46–7.42 (*m*, 3H), 7.33–7.32 (*m*, 1H), 7.26–7.24 (*m*, 1H), 7.02–7.00 (*m*, 1H), 3.89 (*s*, 3H); ^13^C NMR (125 MHz, CDCl_3_) *δ* 160.2, 157.5, 150.2, 140.6, 139.5, 130.2, 129.1, 128.8, 127.2, 119.7, 117.3, 114.3, 113.1, 55.5.

## 6. Refinement

Crystal data, data collection and structure refinement details are summarized in Table 2 ▸. All H atoms were positioned geometrically with C—H = 0.93–0.98 Å and refined as riding atoms. The constraint *U*_iso_(H) = 1.2*U*_eq_ (C) or 1.5*U*_eq_(C_Me_) was applied in all cases.

## Supplementary Material

Crystal structure: contains datablock(s) I. DOI: 10.1107/S2056989022007812/pk2666sup1.cif

Structure factors: contains datablock(s) I. DOI: 10.1107/S2056989022007812/pk2666Isup2.hkl

Supporting information file. DOI: 10.1107/S2056989022007812/pk2666Isup3.cml

CCDC reference: 2194417

Additional supporting information:  crystallographic information; 3D view; checkCIF report

## Figures and Tables

**Table 1:** Hydrogen-bond geometry (Å, °) *Cg*2 and *Cg*3 are the centroids of the C6–C11 and C12–C17 rings, respectively.

*D*—H⋯*A*	*D*—H	H⋯*A*	*D*⋯*A*	*D*—H⋯*A*
C7—H7⋯N1	0.93	2.49	2.8025 (13)	100
C14—H14⋯*Cg*2^i^	0.93	2.74	3.5482 (12)	146
C24—H24*A*⋯*Cg*3^ii^	0.93	2.81	3.6787 (13)	150

Symmetry codes: (i) 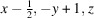 ; (ii) 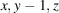 .

**Table 2:** Experimental details

Crystal data	
Chemical formula	C_24_H_19_NO
*M*_r_	337.40
Crystal system, space group	Monoclinic, *I*2/*a*
Temperature (K)	200
*a*, *b*, *c* (Å)	18.6588 (2), 5.4739 (1), 35.5689 (5)
β (°)	100.729 (1)
*V* (Å^3^)	3569.37 (9)
*Z*	8
Radiation type	Cu *K*α
μ (mm^−1^)	0.59
Crystal size (mm)	0.15 × 0.11 × 0.1

Data collection	
Diffractometer	XtaLAB AFC12 (RINC): Kappa single
Absorption correction	Multi-scan (*CrysAlis PRO*; Rigaku OD, 2017 ▸)
*T*_min_, *T*_max_	0.747, 1.000
No. of measured, independent and observed [*I* > 2σ(*I*)] reflections	8525, 3417, 3189
*R*_int_	0.016
(sin θ/λ)_max_ (Å^−1^)	0.615

Refinement	
*R*[*F* ^2^ > 2σ(*F* ^2^)], *wR*(*F* ^2^), *S*	0.034, 0.099, 1.00
No. of reflections	3417
No. of parameters	237
H-atom treatment	H-atom parameters constrained
Δρ_max_, Δρ_min_ (e Å^−3^)	0.19, −0.15

Computer programs: *CrysAlis PRO* (Rigaku OD, 2017 ▸), *SHELXT2014/5* (Sheldrick, 2015*a* ▸), *SHELXL2017/1* (Sheldrick, 2015*b* ▸) and *OLEX2* (Dolomanov *et al.*, 2009 ▸).

## supplementary crystallographic information

### Crystal data

**Table:**

C_24_H_19_NO	*F*(000) = 1424
*M**_r_* = 337.40	*D*_x_ = 1.256 Mg m^−^^3^
Monoclinic, *I*2/*a*	Cu *K*α radiation, λ = 1.54184 Å
*a* = 18.6588 (2) Å	Cell parameters from 6287 reflections
*b* = 5.4739 (1) Å	θ = 2.6–71.4°
*c* = 35.5689 (5) Å	µ = 0.59 mm^−^^1^
β = 100.729 (1)°	*T* = 200 K
*V* = 3569.37 (9) Å^3^	Block, clear light colourless
*Z* = 8	0.15 × 0.11 × 0.1 mm

### Data collection

**Table:**

XtaLAB AFC12 (RINC): Kappa single diffractometer	3417 independent reflections
Radiation source: Rotating-anode X-ray tube, Rigaku (Cu) X-ray Source	3189 reflections with *I* > 2σ(*I*)
Mirror monochromator	*R*_int_ = 0.016
ω scans	θ_max_ = 71.5°, θ_min_ = 2.5°
Absorption correction: multi-scan (CrysAlisPro; Rigaku OD, 2017)	*h* = −20→22
*T*_min_ = 0.747, *T*_max_ = 1.000	*k* = −4→6
8525 measured reflections	*l* = −42→43

### Refinement

**Table:**

Refinement on *F*^2^	Hydrogen site location: inferred from neighbouring sites
Least-squares matrix: full	H-atom parameters constrained
*R*[*F*^2^ > 2σ(*F*^2^)] = 0.034	*w* = 1/[σ^2^(*F*_o_^2^) + (0.0557*P*)^2^ + 1.7292*P*] where *P* = (*F*_o_^2^ + 2*F*_c_^2^)/3
*wR*(*F*^2^) = 0.099	(Δ/σ)_max_ = 0.001
*S* = 1.00	Δρ_max_ = 0.19 e Å^−^^3^
3417 reflections	Δρ_min_ = −0.15 e Å^−^^3^
237 parameters	Extinction correction: SHELXL-2017/1 (Sheldrick 2015b), Fc^*^=kFc[1+0.001xFc^2^λ^3^/sin(2θ)]^-1/4^
0 restraints	Extinction coefficient: 0.00128 (9)
Primary atom site location: dual	

### Special details

**Table:**

Geometry. All esds (except the esd in the dihedral angle between two l.s. planes) are estimated using the full covariance matrix. The cell esds are taken into account individually in the estimation of esds in distances, angles and torsion angles; correlations between esds in cell parameters are only used when they are defined by crystal symmetry. An approximate (isotropic) treatment of cell esds is used for estimating esds involving l.s. planes.

### Fractional atomic coordinates and isotropic or equivalent isotropic displacement parameters (Å^2^)

**Table:**

	*x*	*y*	*z*	*U*_iso_*/*U*_eq_	
O1	0.31876 (5)	0.10179 (16)	0.43410 (2)	0.0435 (2)	
N1	0.56039 (4)	0.98962 (16)	0.36511 (2)	0.0280 (2)	
C1	0.52794 (5)	1.00634 (19)	0.39576 (3)	0.0275 (2)	
C2	0.46613 (5)	0.87064 (19)	0.39895 (3)	0.0295 (2)	
H2	0.445343	0.884525	0.420676	0.035*	
C3	0.43587 (5)	0.71485 (19)	0.36952 (3)	0.0282 (2)	
C4	0.46901 (5)	0.7006 (2)	0.33766 (3)	0.0296 (2)	
H4	0.449647	0.600015	0.317211	0.036*	
C5	0.53147 (5)	0.83820 (19)	0.33658 (3)	0.0276 (2)	
C6	0.57027 (5)	0.8247 (2)	0.30363 (3)	0.0283 (2)	
C7	0.62006 (6)	1.0059 (2)	0.29844 (3)	0.0349 (3)	
H7	0.628023	1.137188	0.315289	0.042*	
C8	0.65777 (7)	0.9923 (2)	0.26844 (3)	0.0416 (3)	
H8	0.690790	1.114477	0.265319	0.050*	
C9	0.64673 (6)	0.7985 (2)	0.24310 (3)	0.0409 (3)	
H9	0.672523	0.789083	0.223154	0.049*	
C10	0.59708 (6)	0.6193 (2)	0.24767 (3)	0.0398 (3)	
H10	0.589029	0.489378	0.230547	0.048*	
C11	0.55906 (6)	0.6315 (2)	0.27768 (3)	0.0349 (3)	
H11	0.525773	0.509472	0.280488	0.042*	
C12	0.37016 (5)	0.5655 (2)	0.37171 (3)	0.0288 (2)	
C13	0.30860 (6)	0.5772 (2)	0.34262 (3)	0.0355 (3)	
H13	0.307468	0.684207	0.322203	0.043*	
C14	0.24943 (6)	0.4289 (2)	0.34437 (3)	0.0393 (3)	
H14	0.208289	0.439001	0.325132	0.047*	
C15	0.25003 (6)	0.2652 (2)	0.37419 (3)	0.0361 (3)	
H15	0.210233	0.163835	0.374717	0.043*	
C16	0.31115 (6)	0.2552 (2)	0.40329 (3)	0.0320 (2)	
C17	0.37049 (5)	0.4078 (2)	0.40215 (3)	0.0301 (2)	
H17	0.410674	0.403553	0.422021	0.036*	
C18	0.56109 (5)	1.17960 (19)	0.42620 (3)	0.0289 (2)	
C19	0.60256 (6)	1.3753 (2)	0.41768 (3)	0.0358 (3)	
H19	0.609761	1.397731	0.392735	0.043*	
C20	0.63323 (7)	1.5371 (2)	0.44594 (4)	0.0458 (3)	
H20	0.661015	1.666984	0.439753	0.055*	
C21	0.62333 (7)	1.5093 (2)	0.48313 (4)	0.0467 (3)	
H21	0.643968	1.619274	0.501995	0.056*	
C22	0.58241 (8)	1.3160 (3)	0.49179 (4)	0.0542 (4)	
H22	0.574972	1.295836	0.516738	0.065*	
C23	0.55208 (7)	1.1507 (3)	0.46386 (3)	0.0456 (3)	
H23	0.525391	1.018874	0.470363	0.055*	
C24	0.26481 (7)	−0.0842 (2)	0.43386 (4)	0.0459 (3)	
H24A	0.261990	−0.182605	0.411288	0.069*	
H24B	0.218282	−0.009630	0.433972	0.069*	
H24C	0.277951	−0.185138	0.456145	0.069*	

### Atomic displacement parameters (Å^2^)

**Table:**

	*U*^11^	*U*^22^	*U*^33^	*U*^12^	*U*^13^	*U*^23^
O1	0.0405 (5)	0.0415 (5)	0.0487 (5)	−0.0114 (4)	0.0090 (4)	0.0073 (4)
N1	0.0251 (4)	0.0288 (4)	0.0301 (4)	−0.0006 (3)	0.0050 (3)	0.0004 (3)
C1	0.0244 (5)	0.0279 (5)	0.0301 (5)	0.0010 (4)	0.0045 (4)	0.0005 (4)
C2	0.0263 (5)	0.0325 (5)	0.0305 (5)	−0.0010 (4)	0.0076 (4)	−0.0012 (4)
C3	0.0227 (5)	0.0294 (5)	0.0320 (5)	0.0006 (4)	0.0041 (4)	0.0009 (4)
C4	0.0261 (5)	0.0328 (5)	0.0292 (5)	−0.0019 (4)	0.0032 (4)	−0.0022 (4)
C5	0.0246 (5)	0.0286 (5)	0.0287 (5)	0.0019 (4)	0.0029 (4)	0.0024 (4)
C6	0.0239 (5)	0.0326 (5)	0.0274 (5)	0.0028 (4)	0.0025 (4)	0.0038 (4)
C7	0.0360 (6)	0.0339 (6)	0.0355 (6)	−0.0019 (5)	0.0087 (4)	0.0019 (5)
C8	0.0399 (6)	0.0449 (7)	0.0431 (6)	−0.0037 (5)	0.0156 (5)	0.0089 (5)
C9	0.0386 (6)	0.0546 (7)	0.0320 (6)	0.0077 (5)	0.0134 (5)	0.0072 (5)
C10	0.0369 (6)	0.0495 (7)	0.0333 (6)	0.0034 (5)	0.0072 (5)	−0.0070 (5)
C11	0.0301 (5)	0.0401 (6)	0.0347 (6)	−0.0027 (5)	0.0062 (4)	−0.0032 (5)
C12	0.0235 (5)	0.0309 (5)	0.0330 (5)	−0.0011 (4)	0.0080 (4)	−0.0063 (4)
C13	0.0294 (5)	0.0439 (6)	0.0328 (5)	−0.0032 (5)	0.0049 (4)	−0.0008 (5)
C14	0.0260 (5)	0.0517 (7)	0.0383 (6)	−0.0049 (5)	0.0009 (4)	−0.0056 (5)
C15	0.0254 (5)	0.0401 (6)	0.0442 (6)	−0.0085 (4)	0.0101 (4)	−0.0097 (5)
C16	0.0300 (5)	0.0310 (5)	0.0369 (5)	−0.0016 (4)	0.0114 (4)	−0.0045 (4)
C17	0.0235 (5)	0.0324 (5)	0.0340 (5)	−0.0012 (4)	0.0042 (4)	−0.0045 (4)
C18	0.0244 (5)	0.0291 (5)	0.0330 (5)	0.0011 (4)	0.0051 (4)	−0.0020 (4)
C19	0.0394 (6)	0.0317 (6)	0.0374 (6)	−0.0038 (5)	0.0097 (5)	−0.0009 (5)
C20	0.0525 (7)	0.0334 (6)	0.0522 (7)	−0.0136 (5)	0.0111 (6)	−0.0051 (5)
C21	0.0507 (7)	0.0425 (7)	0.0453 (7)	−0.0100 (6)	0.0047 (5)	−0.0145 (6)
C22	0.0642 (9)	0.0656 (9)	0.0341 (6)	−0.0236 (7)	0.0127 (6)	−0.0114 (6)
C23	0.0508 (7)	0.0512 (7)	0.0363 (6)	−0.0227 (6)	0.0118 (5)	−0.0058 (5)
C24	0.0394 (6)	0.0326 (6)	0.0703 (9)	−0.0040 (5)	0.0224 (6)	0.0034 (6)

### Geometric parameters (Å, º)

**Table:**

O1—C16	1.3668 (14)	C12—C13	1.3970 (14)
O1—C24	1.4306 (14)	C12—C17	1.3839 (15)
N1—C1	1.3452 (13)	C13—H13	0.9300
N1—C5	1.3432 (13)	C13—C14	1.3811 (16)
C1—C2	1.3940 (14)	C14—H14	0.9300
C1—C18	1.4849 (14)	C14—C15	1.3868 (17)
C2—H2	0.9300	C15—H15	0.9300
C2—C3	1.3864 (14)	C15—C16	1.3913 (16)
C3—C4	1.3905 (14)	C16—C17	1.3936 (15)
C3—C12	1.4879 (14)	C17—H17	0.9300
C4—H4	0.9300	C18—C19	1.3876 (15)
C4—C5	1.3941 (14)	C18—C23	1.3897 (15)
C5—C6	1.4897 (14)	C19—H19	0.9300
C6—C7	1.3946 (15)	C19—C20	1.3812 (17)
C6—C11	1.3934 (15)	C20—H20	0.9300
C7—H7	0.9300	C20—C21	1.3778 (19)
C7—C8	1.3852 (16)	C21—H21	0.9300
C8—H8	0.9300	C21—C22	1.3730 (19)
C8—C9	1.3820 (18)	C22—H22	0.9300
C9—H9	0.9300	C22—C23	1.3845 (18)
C9—C10	1.3796 (18)	C23—H23	0.9300
C10—H10	0.9300	C24—H24A	0.9600
C10—C11	1.3890 (15)	C24—H24B	0.9600
C11—H11	0.9300	C24—H24C	0.9600
			
C16—O1—C24	117.66 (9)	C14—C13—C12	119.56 (11)
C5—N1—C1	118.45 (9)	C14—C13—H13	120.2
N1—C1—C2	122.25 (9)	C13—C14—H14	119.3
N1—C1—C18	116.44 (9)	C13—C14—C15	121.44 (10)
C2—C1—C18	121.31 (9)	C15—C14—H14	119.3
C1—C2—H2	120.2	C14—C15—H15	120.5
C3—C2—C1	119.54 (9)	C14—C15—C16	118.90 (10)
C3—C2—H2	120.2	C16—C15—H15	120.5
C2—C3—C4	118.00 (9)	O1—C16—C15	124.70 (10)
C2—C3—C12	121.48 (9)	O1—C16—C17	115.27 (9)
C4—C3—C12	120.51 (9)	C15—C16—C17	120.02 (10)
C3—C4—H4	120.2	C12—C17—C16	120.58 (10)
C3—C4—C5	119.54 (9)	C12—C17—H17	119.7
C5—C4—H4	120.2	C16—C17—H17	119.7
N1—C5—C4	122.19 (9)	C19—C18—C1	120.49 (10)
N1—C5—C6	116.07 (9)	C19—C18—C23	118.09 (10)
C4—C5—C6	121.73 (9)	C23—C18—C1	121.41 (10)
C7—C6—C5	120.13 (10)	C18—C19—H19	119.7
C11—C6—C5	121.59 (10)	C20—C19—C18	120.57 (11)
C11—C6—C7	118.28 (10)	C20—C19—H19	119.7
C6—C7—H7	119.7	C19—C20—H20	119.5
C8—C7—C6	120.67 (11)	C21—C20—C19	121.08 (12)
C8—C7—H7	119.7	C21—C20—H20	119.5
C7—C8—H8	119.7	C20—C21—H21	120.7
C9—C8—C7	120.54 (11)	C22—C21—C20	118.70 (11)
C9—C8—H8	119.7	C22—C21—H21	120.7
C8—C9—H9	120.3	C21—C22—H22	119.6
C10—C9—C8	119.40 (10)	C21—C22—C23	120.85 (12)
C10—C9—H9	120.3	C23—C22—H22	119.6
C9—C10—H10	119.8	C18—C23—H23	119.7
C9—C10—C11	120.43 (11)	C22—C23—C18	120.69 (12)
C11—C10—H10	119.8	C22—C23—H23	119.7
C6—C11—H11	119.7	O1—C24—H24A	109.5
C10—C11—C6	120.69 (11)	O1—C24—H24B	109.5
C10—C11—H11	119.7	O1—C24—H24C	109.5
C13—C12—C3	120.52 (10)	H24A—C24—H24B	109.5
C17—C12—C3	120.01 (9)	H24A—C24—H24C	109.5
C17—C12—C13	119.44 (10)	H24B—C24—H24C	109.5
C12—C13—H13	120.2		
			
O1—C16—C17—C12	177.88 (9)	C5—N1—C1—C18	−178.87 (9)
N1—C1—C2—C3	−0.90 (15)	C5—C6—C7—C8	178.44 (10)
N1—C1—C18—C19	24.21 (14)	C5—C6—C11—C10	−178.45 (10)
N1—C1—C18—C23	−155.42 (11)	C6—C7—C8—C9	−0.03 (18)
N1—C5—C6—C7	−16.90 (14)	C7—C6—C11—C10	0.57 (16)
N1—C5—C6—C11	162.10 (10)	C7—C8—C9—C10	0.69 (18)
C1—N1—C5—C4	0.54 (15)	C8—C9—C10—C11	−0.70 (18)
C1—N1—C5—C6	−179.20 (9)	C9—C10—C11—C6	0.07 (17)
C1—C2—C3—C4	0.02 (15)	C11—C6—C7—C8	−0.59 (16)
C1—C2—C3—C12	179.70 (9)	C12—C3—C4—C5	−178.60 (9)
C1—C18—C19—C20	179.78 (11)	C12—C13—C14—C15	−0.93 (18)
C1—C18—C23—C22	−179.00 (12)	C13—C12—C17—C16	2.26 (16)
C2—C1—C18—C19	−155.29 (10)	C13—C14—C15—C16	1.44 (18)
C2—C1—C18—C23	25.08 (16)	C14—C15—C16—O1	−179.69 (10)
C2—C3—C4—C5	1.09 (15)	C14—C15—C16—C17	−0.10 (16)
C2—C3—C12—C13	125.72 (11)	C15—C16—C17—C12	−1.75 (16)
C2—C3—C12—C17	−56.26 (14)	C17—C12—C13—C14	−0.93 (16)
C3—C4—C5—N1	−1.42 (15)	C18—C1—C2—C3	178.57 (9)
C3—C4—C5—C6	178.32 (9)	C18—C19—C20—C21	−0.2 (2)
C3—C12—C13—C14	177.10 (10)	C19—C18—C23—C22	1.37 (19)
C3—C12—C17—C16	−175.78 (9)	C19—C20—C21—C22	0.3 (2)
C4—C3—C12—C13	−54.60 (14)	C20—C21—C22—C23	0.5 (2)
C4—C3—C12—C17	123.41 (11)	C21—C22—C23—C18	−1.4 (2)
C4—C5—C6—C7	163.35 (10)	C23—C18—C19—C20	−0.59 (17)
C4—C5—C6—C11	−17.65 (15)	C24—O1—C16—C15	9.02 (16)
C5—N1—C1—C2	0.62 (15)	C24—O1—C16—C17	−170.59 (10)

### Hydrogen-bond geometry (Å, º)

*Cg*2 and *Cg*3 are the centroids of the C6–C11 and C12–C17 rings, respectively.

**Table:**

*D*—H···*A*	*D*—H	H···*A*	*D*···*A*	*D*—H···*A*
C7—H7···N1	0.93	2.49	2.8025 (13)	100
C14—H14···*Cg*2^i^	0.93	2.74	3.5482 (12)	146
C24—H24*A*···*Cg*3^ii^	0.93	2.81	3.6787 (13)	150

Symmetry codes: (i) *x*−1/2, −*y*+1, *z*; (ii) *x*, *y*−1, *z*.
